# CLINICAL AND HISTOLOGICAL COMPARISON OF CELIAC DISEASE AND DERMATITIS HERPETIFORMIS IN MALE PATIENTS: A TEN-YEAR RETROSPECTIVE STUDY

**DOI:** 10.1590/S0004-2803.24612025-016

**Published:** 2025-07-21

**Authors:** Lorete Maria da Silva KOTZE, Eloisa Medeiros NISIHARA, Luiz Roberto KOTZE, Renato NISIHARA

**Affiliations:** 1 Universidade Federal do Paraná, Curitiba, PR, Brasil.; 2 Faculdade Evangélica Mackenzie do Paraná, Curitiba, PR, Brasil.; 3 Universidade Positivo, Curitiba, PR, Brasil.

**Keywords:** Celiac disease, dermatitis herpetiformis, male, symptoms, Doença celíaca, dermatite herpetiforme, masculino, sintomas

## Abstract

**Background:**

Gluten - related diseases, such as celiac disease (CD) and dermatitis herpetiformis (DH) are autoimmune conditions triggered by gluten intolerance. CD manifests with a broad spectrum of clinical symptoms, both intestinal and extraintestinal, while DH is a cutaneous manifestation associated with CD. The clinical manifestations of both CD and DH can vary between men and women.

**Objective:**

This study aimed to describe the clinical profiles and histological findings at the time of diagnosis in men with CD and DH, comparing the differences and similarities between the findings.

**Methods:**

This retrospective study included male patients diagnosed with CD or DH from a specialized private clinic in Curitiba, Brazil. The study involved a review of patients’ clinical charts and was carried out over a ten-year period, from January 2014 to January 2024. CD diagnosis was based on positive serological tests and duodenal biopsies graded by Marsh classification. All patients had DH diagnosis was confirmed through clinical assessment and direct immunofluorescence on skin biopsies before CD diagnosis. All patients were ingesting gluten.

**Results:**

The study analyzed 75 male patients, 57 with CD and 18 with DH. Diarrhea was significantly more prevalent in CD patients, while osteoporosis was exclusively observed in the CD group. Mild enteropathy (Marsh I or Marsh II), accounting for 34.7%, was more commonly associated with DH. In both groups, Marsh III predominated, representing 65.3% of cases. Men with CD and DH displayed similar symptoms.

**Conclusion:**

There were no significant differences in clinical and histological findings between male patients with CD and DH, apart from a higher incidence of diarrhea in CD patients. Duodenal biopsies are recommended for all DH patients.

## HIGHLIGHTS

• Celiac disease and dermatitis herpetiformis shared genetic background and are frequently underdiagnosed.

• Diagnoses of mild enteropathy in both conditions have increased in recent years due to better awareness and improved serological testing.

• Women are more affected and studied. There are few studies in men.

• Overall, clinical presentations were similar between the two diseases.

## RESUMO

**Contexto:**

Doenças relacionadas ao glúten, como a doença celíaca (DC) e a dermatite herpetiforme (DH), são condições autoimunes desencadeadas pela intolerância ao glúten. A DC apresenta um amplo espectro de sintomas, tanto intestinais quanto extraintestinais, enquanto a DH é uma manifestação cutânea frequentemente associada à DC. As manifestações clínicas podem variar entre homens e mulheres.

**Objetivo:**

Descrever os perfis clínicos e achados histológicos no momento do diagnóstico em homens com DC e DH, comparando suas semelhanças e diferenças.

**Métodos:**

Estudo retrospectivo realizado em uma clínica privada especializada em Curitiba, Brasil, envolvendo pacientes do sexo masculino diagnosticados com DC ou DH entre janeiro de 2014 e janeiro de 2024. O diagnóstico de DC foi feito com base em testes sorológicos positivos e biópsias duodenais classificadas de acordo com a classificação de Marsh. O diagnóstico de DH foi confirmado por avaliação clínica e imunofluorescência direta em biópsias de pele, antes da confirmação de DC. Todos os pacientes consumiam glúten.

**Resultados:**

Foram analisados 75 pacientes do sexo masculino, 57 com DC e 18 com DH. Diarreia foi significativamente mais comum nos pacientes com DC, e a osteoporose foi observada exclusivamente nesse grupo. Enteropatia leve (Marsh I ou II) foi mais frequente na DH, sendo presente em 34,7% dos casos, enquanto Marsh III predominou em ambos os grupos (65,3%). Os homens com DC e DH apresentaram sintomas similares.

**Conclusão:**

Não foram observadas diferenças clínicas e histológicas significativas entre homens com DC e DH, exceto pela maior prevalência de diarreia nos pacientes com DC. Recomenda-se a realização de biópsias duodenais em todos os pacientes com DH.

## INTRODUCTION

The Oslo definitions for gluten-related diseases classify these disorders based on their pathogenesis into three categories: autoimmune (such as celiac disease, dermatitis herpetiformis, and gluten ataxia), allergic (wheat allergy), and non-autoimmune, non-allergic (non-celiac gluten sensitivity)^1^. Celiac disease (CD) is a chronic, immune-mediated condition that occurs in genetically predisposed individuals due to an abnormal immune response to gluten ingestion^1^. CD presents with a wide range of clinical symptoms, both extraintestinal and systemic, often acting as a “chameleon.” The incidence of CD is rising, with a global seroprevalence of 1.45% and 0.7% of biopsy-confirmed cases. The female-to-male ratio is approximately 3:1^2^ ^,^ ^3^. Dermatitis herpetiformis (DH) is a relapsing immune-mediated chronic disorder caused also by gluten intolerance and is considered an extraintestinal manifestation of CD^4^ ^,^ ^2^. It is characterized by severe pruritic papulovesicles or excoriated papules on extensor surfaces, buttocks, scalp and nuchal area. Some studies report a proportion male/female 1.5: 1-2.1 with onset about 40.1 years for men The global prevalence is estimated in 11.2 to 75.3 per 100.000 persons^5^. The incidence of DH is decreasing contemporarily^6^. Although the clinical presentation of DH is well-known to many dermatologists, it is not readily suspected or easily recognized in primary care settings^4^.

Both DH and CD share a similar genetic background, with a strong association to human leukocyte antigen (HLA) DQ2/DQ8, and they frequently occur within the same families. As with CD, the primary treatment for DH is a lifelong gluten-free diet (GFD), which helps to improve both the skin rash and the small-bowel mucosal changes^7^.

Laanamanen et al. demonstrated that untreated patients with DH experience gastrointestinal symptoms similar to those of CD. However, the presence of gastrointestinal symptoms at diagnosis is not linked to the severity of the skin symptoms or the clinical resolution of DH on a gluten-free diet (GFD) and these symptoms should not be overlooked, as they significantly affect the patient’s quality of life^8^.

The clinical manifestations of both CD and DH can differ between sexes, with men often experiencing more severe forms and a higher incidence of malab­sorption-related complications^9^. However, as these conditions are more prevalent in women, research has predominantly focused on female-specific findings^8^. Despite population-based studies suggesting that men report fewer diseases and limitations in daily activities, male mortality rates remain consistently higher across all age groups^10^. A critical factor contributing to this disparity is men’s tendency to delay seeking medical attention, often responding only to severe symptoms^11^. This reluctance is influenced by a complex interplay of biological, psychosocial, and social factors, including traditional gender roles. Additionally, lower adherence to medical treatment among men further exacerbates differences in health outcomes^11^. Given these challenges, there is a pressing need for further research focused on men’s health, particularly in understanding disease presentation, healthcare-seeking behaviors, and strategies to improve early diagnosis and adherence to treatment in male patients.

Studies examining the clinical characteristics of male patients with CD and DH remain limited. This study aimed to characterize the clinical profiles and histological findings at the time of diagnosis in men with these conditions, emphasizing the identification and comparison of key findings.

## METHODS

### Design and ethical issues

This retrospective study was approved by the local Research Ethics Committee under protocol 6.566.826 and was conducted in accordance with the Good Clinical Practice Guidelines and the Declaration of Helsinki. Informed consent for participation was obtained from all patients. The study involved a review of patients’ clinical charts and was carried out over a ten-year period, from January 2014 to January 2024. All patients were treated by the same physician at a reference Gastroenterology medical office in Curitiba, Paraná, Brazil.

### Patients recruitment

We included male patients diagnosed with CD based on signs and symptoms, as well as positive serological findings of autoantibodies anti-endomysial-IgA and/or anti-transglutaminase antibodies-IgA, following determination of serum levels IgA^2^ ^,^ ^3^.

In addition, we included male patients with DH without a prior diagnosis of CD. The diagnosis of DH was established by history, physical examination of the eruptions and confirmed by skin biopsies performed by direct immunofluorescence of unaffected skin in close proximity to an active lesion. This reveals pathognomonic granular IgA deposits at the dermo-epidermal junction^5^ ^,^ ^6^.

All the patients underwent upper gastrointestinal endoscopy^3^ and histopathological findings in duodenal biopsies were availed according to the Marsh classification^12^.

All patients were on a gluten-containing diet. Female patients and those with incomplete records were excluded from the study.

### Data collection

 All data were collected at diagnosis. Clinical charts were reviewed, and data included were demographic and anthropometric parameters such as age, age at symptoms onset, diagnostic delay, BMI, gastrointestinal symptoms, weight loss, and anemia. Bone disease was assessed using dual-energy X-ray absorptiometry (DXA) at the lateral distal femur and anterior-posterior spine^2^ ^,^ ^3^. HLA DQ2/DQ8 was performed in cases of diagnosis doubt or discrepancies in the results^2^ ^,^ ^13^. Familial gluten-related disorders and other previously diagnosed comorbidities were self-reported.

### Data analysis

Data was collected in frequency tables. Statistical analyzes were performed using the SPSS program. 17.0. The Kolmogorov-Smirnov and Shapiro-Wilk tests were used to assess data normality. Continuous variables were expressed as median and interquartile range (IQR), and were compared using the non-parametric Mann-Whitney test. Categorical variables were expressed in percentages and compared using the Fisher’s exact test or chi-squared, as appropriate. *P* values less than 0.05 were considered statistically significant.

## RESULTS

The cohort consisted of 75 Caucasian male patients, being 57 with CD median age 38.0 (IQR=19-49) years at diagnosis and 18 with DH without previous diagnosis of CD, median age at diagnosis 39 years (IQR=22-47 years).

Regarding BMI, only patients with CD exhibited thinness, with 12.0% affected. Adequate BMD was found in 56.9% of CD patients and 64.7% of those with DH. Overweight was observed in 24.8% of CD patients and 35.3% of DH patients.

Table 1 presents clinical signs and symptoms of the patients studied at diagnosis. Diarrhea was significantly more common in patients with CD compared to those with DH. The occurrence of anemia, weight loss, and osteopenia was similar between CD and DH patients, while osteoporosis was observed exclusively in CD patients.

**TABLE 1:** Signs and symptoms at diagnosis of male patients with celiac disease (n=57) or dermatitis herpetiforme (n=18) studied.

Sign or symptom	Celiac disease n (%)	Dermatitis herpetiforme n (%)	***P* value**
Abdominal distention	50/57 (88.6)	13/17 (76.5)	0.43
Flatulence	46/57 (84.7)	13/17 (76.5)	0.41
Diarrhea	43/57 (75.8)	2/17 (11.8)	<0.0001
Gastroesophagic reflux	33/57 (62.4)	9/17 (52.9)	0.57
Abdominal pain	31/57 (54.0)	5/17 (29.4)	0.27
Aphthous ulcer	20/57 (36.8)	5/17 (29.4)	0.77
Epigastric pain	16/57 (30.7)	5/17 (29.4)	0.55
Nausea	11/57 (18.8)	1/16 (6.2)	0.27
Mal digestion	10/57 (17.7)	3/15 (20.0)	0.43
Constipation	6/57 (9.8)	4/17 (23.5)	0.22
Vomit	2/57 (3.7)	2/16 (12.5)	0.21
Weight loss	25/57 (75.6)	6/9 (55.5)	0.28
Anemia	13/57 (30.4)	3/16 (18.7)	0.90
Femur osteopenia	13/57 (22.8)	3/11 (27.3)	0.87
Femur osteoporosis	3/57 (5.3)	0/11	0.33
Spine osteopenia	13/57 (22.8)	3/11 (27.3)	0.87
Spine osteoporosis	9/57 (15.8)	0/11	0.24

Table 2 shows the presence of DQ2 and/or DQ8, with no significant difference between CD and DH patients. It also presents the duodenal biopsy findings, which revealed no notable differences between the two conditions. Mild enteropathy (Marsh I or Marsh II), accounting for 34.7%, was more commonly associated with DH. In both groups, Marsh III predominated, representing 65.3% of cases.

**TABLE 2:** Histological findings in duodenal biopsy according to Marsh classification and HLA DQ2/DQ8 research in the cases studied.

	Celiac disease n (%)	Dermatitis herpetiforme n (%)	***P* value**
Marsh I	5/57 (8.8)	3/18 (16.7)	0.38
Marsh II	12/57 (21.0)	6/18 (33.3)	0.34
Marsh III	40/57 (70.2)	9/18 (50.0)	0.15
HLA DQ2 + DQ8 -	15/22 (68.2)	8/10 (80.0)	0.20
HLA DQ2 - DQ8 +	4/22 (18.1)	1/10 (10.0)	0.42
HLA DQ2 + DQ8 +	3/22 (13.7)	1/10 (10.0)	0.98

Regarding family history, nine CD patients (15.8%) reported relatives with CD, including three mothers, three sisters, one father, one brother, and one son. Among DH patients, four cases (22.2%) reported affected relatives, with three sisters having CD and one sister with DH. Comorbidities such as anxiety, depression, hypothyroidism, and migraines were refereed in both conditions.

## DISCUSSION

This study presents clinical data in the Brazilian male population with CD and DH at diagnosis, showing that in both groups the clinical symptoms/signs are similar and both affect equally the duodenal mucosa. Our study also emphasizes the main findings and challenges in diagnosing these disorders within in the gastroenterology office.

There was no difference in the age distribution between the two disorders or in relation to age at diagnosis. Our study is in accordance with the reports that CD is diagnosed at any age^2^ ^,^ ^3^ and DH patients showed the same previous reports concerning age with prevalence in adult^5^ ^,^ ^6^.

About BMI findings, we found thinness exclusively in CD patients, with similar rates of adequate BMI in both disorders. Notably, an expressive number of individuals in both groups were overweight. Cheng et al. reported that American males with CD typically had a normal BMI, with fewer cases of underweight^14^. In our study, patients with CD referred significantly more diarrhea. The others symptoms/signs were very similar. Interesting, oral lesions were described as rare in DH^15^; however in our sample, we found in 29.4 % of our patients and in 36.8 % in CD. These findings can explain the lower BMI in CD patients.

Comparing gastrointestinal complaints in men with CD is difficult, as most studies include both genders. Common symptoms such as diarrhea, bloating, abdominal distension, flatulence, gastroesophageal reflux, abdominal pain, and aphthous ulcers have been frequently reported^2^ ^,^ ^3^ ^,^ ^16^. Previous studies have found GI symptoms in only 20% of patients with DH^4^ ^,^ ^6^. However, our study revealed a higher prevalence of GI complaints in DH patients, closely resembling those seen in CD. It is likely that DH patients, focused on their skin symptoms and reduced quality of life, may be unaware of their underlying GI issues^17^.

Our study detected bone changes in both diseases, which appear to be more related to age than to the extent of duodenal damage. The prevalence of osteoporosis in CD and DH, particularly in men, seems to be underestimated, even though screening is recommended at diagnosis^3^, especially in adults approaching andropause^16^. Despite men generally having higher BMI than women, osteoporosis is more common in men^9^. Bone disorders have been reported in CD^18^, while some authors have denied their presence in DH^19^.

 In relation to duodenal histology and BMI, Garcia-Manzanares et al. demonstrated a correlation between BMI and duodenal Marsh stage in newly diagnosed CD patients, a pattern we also observed in patients with DH^20^. Male patients with CD showed a high prevalence of low BMI, particularly those over 50 years old^21^. Similarly, in DH, low BMI was noted even in younger patients. Galli et al., in a study of 214 patients, reported 42.5% with osteopenia and 17.8% with osteoporosis, highlighting that osteoporosis in CD was significantly associated with age over 45, male gender, low BMI, and Marsh III-findings consistent with our own^22^. Our results also align with those of Walter et al. in the USA^8^, who emphasized that fracture risk is similar in CD and DH. In our study, osteopenia in the femur occurred at nearly the same rate in both CD and DH, but osteoporosis was only present in CD, suggesting that bone disease is more severe in CD. Notably, none of the patients reported fractures at diagnosis.

Family screening for gluten sensitivity is highly recommended for both CD and DH^5^ ^,^ ^23^. In our study, 15.8% of CD patients and 22.2% of DH patients reported gluten-related diseases in their families. These findings highlight the importance of closely monitoring first-degree relatives and confirm the predominance of female cases, as noted by other studies^24^.

Our results showed a predominance of HLA DQ2 positivity, followed by DQ8. The findings from HLA research in our cases of CD and DH confirmed the shared genetic predisposition between these diseases, consistent with previous studies^5^.

Histological findings from duodenal biopsies in our study showed greater severity in CD compared to DH. Notably, 70.2% of CD patients and 50.0% of DH patients were classified as Marsh III at diagnosis, including younger individuals, which is consistent with previous findings^17^. However, these authors reported that at least one-quarter of DH patients had normal villous architecture. In contrast, none of the DH patients in our study presented with normal mucosa in their duodenal biopsies. Mild enteropathy is characterized by a Marsh I or II lesion on small intestinal biopsy without villous atrophy^23^ ^,^ ^16^. In our study, Marsh I was observed in 10.7% of cases and Marsh II in 24.0%, compared to 65.3% with Marsh III, consistent with findings from other authors^25^ ^,^ ^26^. It is noteworthy that diagnoses of mild enteropathy in CD and DH have increased over the past two decades, likely due to heightened awareness and the availability of more sensitive serological tests^25^ ^,^ ^26^. Some studies suggest that mild enteropathy is not necessarily a mild condition and differs only minimally from classical CD/DH with villous atrophy, which supports the recommendation for a GFD in cases of mild enteropathy^25^ ^-^ ^27^. While duodenal biopsies are considered the gold standard for diagnosing CD^2^ ^,^ ^3^ ^,^ ^16^, there is some controversy regarding their necessity in DH^6^. The authors of this study recommend performing biopsies in all DH cases at diagnosis to differentiate from other enteropathies, to detect gastrointestinal complications or malignancies (such as lymphoma, which poses a higher risk), and monitor treatment progress during follow-up^23^ ^,^ ^28^.

The primary treatment for CD and DH is lifelong strict adherence to a GFD^2^ ^,^ ^3^ ^,^ ^16^. In DH, a GFD improves quality of life and offers a favorable long-term prognosis^6^. Dapsone is another treatment option that provides rapid relief from pruritus, often within hours, making it effective for managing acute inflammatory phases. However, it does not affect the underlying enteropathy, IgA deposits, or the risk of lymphoma^5^ ^,^ ^29^.

This study has some limitations due to its retrospective design. Additionally, as it was conducted in a referral private practice, there is a possibility that the patients included may have had more severe or complicated forms of the disease. However, the key strengths of the study lie in the fact that all patients were evaluated by the same team of professionals, following a consistent clinical, laboratory, and histological protocol.

Culturally, Brazilian men are more likely than women to avoid medical appointments and health care, a topic widely addressed in recent studies. Research suggests that traditional masculinity norms in Brazil, as in other cultures^10^ ^,^ ^11^, promote the belief that men must be strong, self-reliant, and refrain from showing vulnerability, which includes seeking medical help^30^. These norms reinforce the perception that visiting a doctor is a sign of weakness or dependency, leading to lower adherence to preventive healthcare and treatments. Consequently, men often delay seeking medical attention, resulting in the aggravation of potentially manageable conditions as CD or DH. To optimize patient support, a multidisciplinary team-including a general practitioner, dermatologist, pediatrician, gastroenterologist, psychologist, and expert dietician-can enhance well-being, improve dietary adherence, to face health related stigmas^31^ and encourage participation in support groups for additional guidance and improve your the quality of life^16^ ^,^ ^32^.

## CONCLUSION

In conclusion, no clinical differences were found between male patients with CD or DH at diagnosis, except for a higher frequency of diarrhea in patients with CD. Duodenal biopsy findings were similar in both groups, with mild or severe disease.

## References

[1] Ludvigsson JF, Leffler DA, Bai JC, Biagi F, Fasano A, Green PH (2013). The Oslo definitions for coeliac disease and related terms. Gut.

[2] Al-Toma A, Volta U, Auricchio R, Castillejo G, Sanders DS, Cellier C (2019). European Society for the Study of Coeliac Disease (ESsCD) guidelines for coeliac disease and other gluten-related disorders. U. Eur. Gastroenterol. J.

[3] Rubio-Tapia A, Hill ID, Semrad C, Kelly CP, Greer KB, Limketkai BN (2023). American College of Gastroenterology Guidelines Update: Diagnosis and management of celiac disease. Am J Gastroenterol.

[4] Reunala T, Hervonen K, Salmi T (2021). Dermatitis Herpetiformis: An update on diagnosis and management. Am J Clin Dermatol.

[5] Nguyen CN, Kim SJ (2021). Dermatitis herpetiformis: An update on diagnosis, disease monitoring, and management. Medicina.

[6] Salmi T, Hervonen K (2020). Current concepts of dermatitis herpetiformis. Acta Derm Venereol.

[7] Sernicola A, Mazzetto R, Tartaglia J, Ciolfi C, Miceli P, Alaibac M (2023). Role of human l eukocyte antigen class II in antibody-mediated skin disorders. Medicina.

[8] Laamanen A, Salmi T, Huhtala H (2024). Gastrointestinal symptoms at diagnosis and during long-term gluten-free diet treatment in dermatitis herpetiformis patients. J Eur Acad Dermatol Venereol.

[9] Walker MD, Williams J, Lewis SK, Bai JC, Lebwohl B, Green PHR (2020). Measurement of forearm bone density by dual energy x-ray absorptiometry increases the prevalence of osteoporosis in men with celiac disease. Clin Gastroenterol Hepatol.

[10] Juel K, Christensen K (2008). Are men seeking medical advice too late? Contacts to general practitioners and hospital admissions in Denmark 2005. J Public Health (Oxf).

[11] Oksuzyan A, Juel K, Vaupel JW, Christensen K (2008). Men: good health and high mortality. Sex differences in health and aging. Aging Clin Exp Res.

[12] Marsh MN (1992). Gluten, major histocompatibility complex, and the small intestine. A molecular and immunobiologic approach to the spectrum of gluten sensitivity (‘celiac sprue’). Gastroenterology.

[13] Husby S, Murray JA, Katzka DA (2019). AGA Clinical practice update on diagnosis and monitoring of celiac disease. Changing utility of serology and histologic measures: Expert review. Gastroenterology.

[14] Cheng J, Brar PS, Lee AR, Green PHR (2010). Body mass index in celiac disease: beneficial effect of a gluten-free diet. J Clin Gastroenterol.

[15] Mania-Końsko A, Szponar E, Dańczak-Pazdrowska A, Bowszyc-Dmochowska M, Pazdrowski J, Wyganowska M (2023). Imunopathological assessment of the oral mucosa in dermatitis herpetiformis. Int J Environ Res Public Health.

[16] Elli L, Leffler D, Cellier C, Lebwohl B, Ciacci C, Schumann M (2024). Guidelines for best practices in monitoring established coeliac disease in adult patients. Nat Rev Gastroenterol Hepatol.

[17] Mansikka E, Salmi T, Kaukinen K, Collin P, Huhtala H, Reunala T (2018). Diagnostic delay in dermatitis herpetiformis in a high-prevalence area. Acta Derm Venereolo.

[18] Volta U, Caio G, Stanghellini V, De Giorgio R (2014). The changing clinical profile of celiac disease: a 15-year experience (1998-2012) in an Italian referral center. BMC Gastroenterol.

[19] Lorinczy K, Juhász M, Csontos Á, Fekete B, Terjék O, Lakatos PL (2013). Does dermatitis herpetiformis result in bone loss as coeliac disease does? A cross sectional study. Rev Esp Enferm Dig.

[20] Garcia-Manzanares A, Tenias JM, Lucendo AJ (2012). Bone mineral density directly correlates with duodenal Marsh stage in new diagnosed adult celiac patients. Scand J Gastroenterol.

[21] Kotze LM, Skare TL, Kotze LR, Nisihara R (2024). Skeletal health assessment in Brazilian men with celiac disease at diagnosis: how important is it?. Arch Gastroenterol.

[22] Galli G, Lahner E, Annibale B (2020). Do alterations in bone mineral density go beyond borders in adults with celiac disease?. Clin Gastroenterol Hepatol.

[23] Kárpáti S (2012). Dermatitis herpetiformis. Clin Dermatol.

[24] Singh P, Arora S, Lal S, Strand TA, Makharia GK (2015). Risk of celiac disease in the first- and second-degree relatives of patients with celiac disease: A systematic review and meta-analysis. Am J Gastroenterol.

[25] Kurppa K, Colin P, Sievänen H, Huhtala H, Mäki M, Kaukinen K (2010). Gastrointestinal symptoms, quality of life and bone mineral density in mild enteropathic coeliac disease: a prospective clinical trial. Scand J Gastroenterol.

[26] Zanini B, Caselani F, Magni A, Turini D, Ferraresi A, Lanzarotto F (2013). Celiac disease with mild enteropathy is not mild disease. Clin Gastroenterol Hepatol.

[27] Leffler D, Vanga R, Mukherjee R (2013). Mild enteropathy celiac disease: a wolf in sheep’s clothing?. Clin Gastroenterol Hepatol.

[28] Grainge MJ, West J, Solaymani-Dodaran M, Card TR, Logan RF (2012). The long-term risk of malignancy following a diagnosis of coeliac disease or dermatitis herpetiformis: a cohort study. Aliment Pharmacol Ther.

[29] Kotze LMDS, Kotze LR, Purim KSM, Nisihara R (2021). The management of dermatitis herpetiformis by the gastroenterologist. A series of cases. Arq Gastroenterol.

[30] Gomes R, Nascimento EF, Araújo FC (2007). Why do men use health services less than women? Explanation by men with low versus higher education. Cad Saude Publica.

[31] Pitt A, Lerigo F, Satherley RM (2024). Health-related stigma and challenges faced by men living with celiac disease: A qualitative analysis. J Acad Nutr Diet.

[32] Mulder CJJ, Elli L, Lebwohl B, Makharia GK, Rostami K, Rubio-Tapia A (2023). Follow-up of celiac disease in adults: “When, what, who, and where”. Nutrients.

